# The Royal Veterinary College

**Published:** 1891-12

**Authors:** 


### THE ROYAL VETERINARY COLLEGE.*

On April 8th, 1791, the meeting was held at which it was determined to found a Veterinary College in London. The Duke of Northumberland was President, and other noblemen and gentlemen subscribed liberally to give effect to the scheme. Charles Vial de St. Bel was appointed Professor to the contemplated institution, and a Committee was formed to carry out the scheme. John Hunter, Mr. Cline, Sir Joseph Banks, Dr. Crawford, and other leading members of the medical profession, gave their assistance, and very full public approbation followed.

A piece of ground covering some acres in St. Pancras was taken on a long lease from the Earl of Camden, and arrangements made for building a lecture theatre, a disssecting room, stabling for fifty horses, and a strong forge. This was only a temporary plan, for St. Bel had much grander ideas of what a college should

* The Veterinary Record, October, 1891.

be ; he had been trained in the Government schools of France, and desired that the English school should be founded on a similar basis. His curriculum would have extended over three years, and all domestic animals would have obtained attention ; but St. Bel's scheme was too large for the private efforts of his subscribers, and too far advanced for the public of his day. The stables, the forge and lecture theatre were, however, completed, and on January 1st, 1792, a course of lectures was commenced. It was not till January 1st, 1793, that the infirmary was opened for patients, and then in a few days every stall was occupied. Ten students had joined the classes, and the anatomy of the horse was studied in the dissecting room. Amongst the students were men who left a mark on the profession, and whose names are worth remembering—they were Messrs. Bloxam, Blaine, R. Laurence, Field, and Bracy Clark.

Unfortunately the money subscribed to found the College was lavishly spent, and the buildings were not completed. Difficulties arose, and subscribers who began by being over-sanguine ended by being disappointed and suspicious. This, says Bracy Clark, "was shortly after followed by an event which for a time seemed likely to terminate the misfortunes of the College, and its existence ; this was no less than the death of our professor." The death of St. Bel took place in August, 1793, and the affairs of the College became gloomy indeed : creditors were pressing, and it seemed at one time that the institution must come to an end—for no one equally qualified could be found to take the place of St. Bel. We are told by Bracy Clark that "the pupils, being left without a teacher, were unable to make any considerable progress in their studies. In this position a very generous offer was made them by several of the most celebrated professors of human anatomy and medicine in London, which was to attend their lectures free of all expense. The lecture rooms of Dr. Fordyce, Dr. Baillie, Mr. Hunter, and Mr. Cruikshank, were now open to them, and most of them, with much industry availed themselves of the offer."

During this interregnum the practice of the College was carried on by the elder pupils, and Mr. Richard Laurence deserves to be remembered as the individual who practically succeeded St. Bel, and remained in charge until the Governing Committee found a successor to the first principal. The financial condition of the school had reached its lowest point, and the Committee were threatened with lawsuits by the creditors. At this juncture a meeting was called, and the Earl of Morton and Lord Heathfield again gave their assistance. John Hunter also gave his influence and money, and the College was resuscitated. In 1794 the directorship was entrusted to Messrs. Moorcroft and Coleman, both of whom were surgeons, but the former had, after studying veterinary science in France, practiced in London as a veterinary surgeon, and attained such celebrity as to bring back confidence in the College at once. For a few weeks the business of the institution was conducted to the great satisfaction of the subscribers—"the deserted College became again filled with horses"—when the sudden resignation of Moorcroft gave a shock to the struggling, and so far, unfortunate institution. Coleman was left as sole professor, and he only withdrew his resignation at the earnest request of his friends, amongst whom Sir Astley Cooper was prominent. Coleman had no practical knowledge of horses or their diseases, but he was clever and energetic, a good human anatomist, and an astute business man, with strong friends in the medical world. Writing in 1798 Mr. Bracy Clark says: "The institution now remained solely to be conducted by Mr. Coleman, from whose abilities and ingenuity the science has everything to expect; he has been able during the three years that he has been professor, not only to maintain the institution in prosperity, but has had the address, with the aid of the Committee, to procure the pecuniary assistance of Government, which now affords the institution a handsome annual supply, and places it beyond the probability of ever being destroyed on this account." In these few years, too, Professor Coleman had obtained for himself the appointment of Principal Veterinary Surgeon to the Army, and was entrusted with the selection of veterinary surgeons for the cavalry.

The College during the first ten years of its existence held a very prominent position—it was constantly before the public for some reason or other. The movement which gave it origin had hardly subsided before the death of its first professor again brought it into notice; then Coleman and Moorcraft's joint professorship and its sudden rupture; next the position given to Coleman as Chief of the Veterinary Department of the Cavalry. The books and pamphlets issued by those connected with the College also had an effect. St. Bel, Moorcroft, and Coleman, all wrote on the horse's foot and shoeing. St. Bel, and the practice he introduced at the College, assisted in reducing the weight of shoes then used, and in checking the practice of seating shoes in the form of a saucer. He also recommended a shoe flat on the foot surface and concave towards the ground. Moorcroft, following an older authority, Osmer, advised a shoe having a good level bearing surface, but not so wide as to press on the sole, to avoid which he had it "seated"—and this form of shoe is the one most commonly used at this day. Moorcroft's essay on shoeing had one good point—he recognised the value of frog-pressure; but to this he sacrificed other necessary principles. He advised parting the soles thin, especially at the angles of the heels. and the use of a thin-heeled shoe. The effect upon the art of horse-shoeing in London, of all the attention given to the subject at the College must have been very great—whether for good or evil is not clear.

From 1791 to 1800 there is evidence of very considerable activity at the College. Students apparently went there with a determination to study, and quite a number of the earlier ones have left some record of work. Blaine, Bracy Clark, White, Percivall, Feron, Laurence, Boardman and all names of men who afterwards contributed to veterinary literature.

Coleman for a time after his appointment was apparently sole professor and teacher. John Percivall was "Assistant Veterinary Surgeon to the Professor" in 1797 and perhaps some years earlier. In 1796 William Sewell, whose father was a farmer in Essex, was apprenticed to Coleman, and in 1799 passed his examination and was appointed Assistant Demonstrator of Anatomy.

The Examining Committee who granted certificates of proficiency to the students consisted entirely of medical men, assisted by Professor Coleman. For the first twenty years of the College, this was perhaps as good an arrangement as could be made. There were few veterinary graduates whose names would have carried much weight on a certificate, but the names of the leading men in medicine and surgery carried great weight, and gave that connection with science which the art then so much needed. Students were taught Anatomy and Scientific principles, which gave them at any rate an immense superiority over the older practitioners with whom they had to compete.

The early teaching at the College, it must be confessed, was very limited. The horse was the only animal referred to, and one professor and an assistant had to do all the practice and teaching. Unless this had been supplemented by the magnaminous permission of leading medical teachers, for veterinary students to attend their lectures gratuitously, our pioneers would have been mere pretenders to scientific knowledge. Upon the ex-collegiate classes depended all their training in chemistry, materia-medica, and physiology.

St. Bel had started the venture with a much more extensive scheme, and with a much higher standard of the necessities of veterinary students. He was an enthusiast. Coleman was a practical man who "cut his clothes according to his cloth," and permitted no veterinary enthusiasm to outrun his judgment. He obtained financial assistance from Government, an official position for himself, and he accepted the medical assistance so kindly offered without any feeling of compunction that the school did not supply within its own wall all the students' wants.

From 1800 to 1825 very little is known of the proceedings of the Veterinary College. There were no periodicals in those days, and the staff very judiciously committed nothing to paper. We only know that the institution progressed quietly, that students entered and studied, that the practice increased, and that the Professor maintained the dignity of his position in a proper manner. In 1820 the Annual Parliamentary Grant of £1000 ceased, but the College was able to run alone, and took no harm from the withdrawal of Treasury support.

Occasional glimpses of the College are obtained from writings of old students. Thus we learn that Mr. Mayer, of Newcastle, was a student in 1811, and on joining had a ticket of admission to all the lectures at a medical school, presented to him by Professor Coleman. From attending these lectures he joined the Westminster Medical Society, and thus got the idea of starting a veterinary medical society. This he with other students founded in 1812, and their weekly meetings were held in Marchmont Street. In the second year Professor Sewell allowed himself to be nominated as President, but Coleman looked askance at the new society. In the course of another year or two the Society was admitted into the College, and Professor Coleman became patron.

In 1817 William Dick entered as a student, and has left us a glimpse of the College in two letters he wrote whilst a student, to his father. The impression is that a student had to depend greatly upon himself; Sewell was occupied by practice, and Coleman was very often away on consultations, or the business of the Army and Ordnance Department which he supervised. Dick does not seem to estimate the diagnostic powers of the staff very highly, and the treatment of animals described by him shows a practice differing very slightly from the empirical art carried on around.

